# Design and Evaluation of a Hybrid Radiofrequency Applicator for Magnetic Resonance Imaging and RF Induced Hyperthermia: Electromagnetic Field Simulations up to 14.0 Tesla and Proof-of-Concept at 7.0 Tesla

**DOI:** 10.1371/journal.pone.0061661

**Published:** 2013-04-22

**Authors:** Lukas Winter, Celal Özerdem, Werner Hoffmann, Davide Santoro, Alexander Müller, Helmar Waiczies, Reiner Seemann, Andreas Graessl, Peter Wust, Thoralf Niendorf

**Affiliations:** 1 Berlin Ultrahigh Field Facility (B.U.F.F.), Max-Delbrueck Center for Molecular Medicine, Berlin, Germany; 2 Metrology in Medicine, Physikalisch Technische Bundesanstalt, Berlin, Germany; 3 Clinic for Radiation Oncology, CVK, Charité Universitätsmedizin Berlin, Germany; 4 Experimental and Clinical Research Center (ECRC), a joint cooperation between the Charité Medical Faculty and the Max-Delbrueck Center for Molecular Medicine, Berlin, Germany; University of Minnesota, United States of America

## Abstract

This work demonstrates the feasibility of a hybrid radiofrequency (RF) applicator that supports magnetic resonance (MR) imaging and MR controlled targeted RF heating at ultrahigh magnetic fields (B_0_≥7.0T). For this purpose a virtual and an experimental configuration of an 8-channel transmit/receive (TX/RX) hybrid RF applicator was designed. For TX/RX bow tie antenna electric dipoles were employed. Electromagnetic field simulations (EMF) were performed to study RF heating versus RF wavelength (frequency range: 64 MHz (1.5T) to 600 MHz (14.0T)). The experimental version of the applicator was implemented at B_0_ = 7.0T. The applicators feasibility for targeted RF heating was evaluated in EMF simulations and in phantom studies. Temperature co-simulations were conducted in phantoms and in a human voxel model. Our results demonstrate that higher frequencies afford a reduction in the size of specific absorption rate (SAR) hotspots. At 7T (298 MHz) the hybrid applicator yielded a 50% iso-contour SAR (iso-SAR-50%) hotspot with a diameter of 43 mm. At 600 MHz an iso-SAR-50% hotspot of 26 mm in diameter was observed. RF power deposition per RF input power was found to increase with B_0_ which makes targeted RF heating more efficient at higher frequencies. The applicator was capable of generating deep-seated temperature hotspots in phantoms. The feasibility of 2D steering of a SAR/temperature hotspot to a target location was demonstrated by the induction of a focal temperature increase (ΔT = 8.1 K) in an off-center region of the phantom. Temperature simulations in the human brain performed at 298 MHz showed a maximum temperature increase to 48.6C for a deep-seated hotspot in the brain with a size of (19×23×32)mm^3^ iso-temperature-90%. The hybrid applicator provided imaging capabilities that facilitate high spatial resolution brain MRI. To conclude, this study outlines the technical underpinnings and demonstrates the basic feasibility of an 8-channel hybrid TX/RX applicator that supports MR imaging, MR thermometry and targeted RF heating in one device.

## Introduction

Magnetic Resonance Imaging (MRI) is of proven diagnostic value with an ever growing number of applications that support interventional procedures and therapies [1]–[4]. MR controlled interventions include localized cell, drug and contrast agent delivery [5], [6], radio frequency (RF) ablation [7], [8] and thermotherapy during regional RF induced hyperthermia [9]–[13] to name a few.

The clinical value of regional RF hyperthermia as an adjunctive therapy to radiotherapy and chemotherapy has been demonstrated [14]–[21]. In current clinical RF hyperthermia practice RF coils are being used for imaging and MR thermometry (MRTh) for spatiotemporal monitoring of temperature and treatment efficacy [22], [23]. While the RF coils used for MR imaging are commonly operated at a frequency of 64 MHz (1.5 T), RF transmission induced heating interventions are achieved with an applicator commonly driven at a frequency of 70–100 MHz [24]. Consequently current clinical implementations require extra hardware retrofitted into the MR suite – notably antennas, amplifiers and frequency filters – which have the trait of driving costs, limiting patient comfort and ease of use and which bear the potential to induce imaging artifacts [25].

Another recognized limitation of current MR guided RF hyperthermia therapies is the RF wavelength used for RF transmission. The RF wavelength is given by the ratio between the phase speed 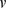 and frequency *f*. This wavelength is shortened by the refractive index 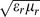, which leads to an effective wavelength 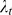 in biological tissue (μ_r_≈1). At 1.5 T the ^1^H spin excitation frequency of 64 MHz results in 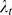 of approximately 60 cm (assuming ε = 60 and muscle tissue). An excitation frequency of *f* = 100 MHz results in 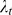 of approximately 38 cm while at 3.0 T (f = 128 MHz) 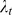 is approximately 30 cm. These wavelengths are relatively long compared to the geometry of a human torso let alone the geometry of the human brain. This constraint limits the spectrum of interventions and therapies using MR guided RF hyperthermia [14] and so suggests that innovations are needed.

At ultrahigh magnetic fields (UHF, B_0_≥7.0 T) the ratio between the wavelength inside the human body and its volume is significantly reduced. Effective wavelengths of approximately 13 cm at 7.0 T or as small as approximately 6 cm at 14.0 T hold the promise to further advance the capabilities of MR controlled RF hyperthermia interventions. Admittedly, the wave length shortening at UHF constitutes a major challenge for imaging due to highly complex interference patterns and non-uniform RF transmission field (B_1_^+^) distributions [26]. This challenge can be addressed by using B_1_^+^ shimming techniques and multi-channel transmit (TX) RF technology [27]–[29]. Multi-channel TX RF technology also provides capabilities for tailoring the electric field E - the component of electro-magnetic fields (EMF) that primarily contributes to RF power deposition - by means of constructive and destructive interferences. E-fields are a major source for tissue heating which is governed by the specific absorption rate (SAR). Realizing the opportunities together with the limitations of current MR guided RF heating procedures this work proposes a novel hybrid applicator that affords diagnostic MR imaging, MR thermometry and targeted RF heating at ultrahigh fields. For this purpose, a multi-channel transceiver (TX/RX) RF coil array that makes use of building blocks comprised of bow tie shaped electric dipole antennas is proposed. Its design and its capability for RF heating are examined in numerical electromagnetic field (EMF) and in temperature simulations. For this purpose RF frequencies ranging from 64 MHz (1.5 T) to 600 MHz (14.0 T) are used. These efforts are paralleled by careful MR safety considerations to meet the RF power deposition constraints given by the IEC guidelines [30]. The feasibility of the proposed hybrid applicator for MR imaging, for spatio-temporally controlled and MRTh monitored localized RF heating is demonstrated. This includes the feasibility of inducing deep-seated SAR and temperature hotspots plus the proof-of-principle of 2D steering of local SAR and temperature hotspots. To meet this goal phantom studies using an RF transmission frequency of 297 MHz are conducted at 7.0 T. EMF and temperature simulations in a human voxel model deduced from a healthy volunteer demonstrate the feasibility of the proposed hybrid setup for targeted RF heating in the human brain. The merits and limitations of the hybrid applicator are discussed and implications for UHF-MR hyperthermia interventions are considered.

## Materials and Methods

### Ethics Statement

All imaging studies were performed after due approval by the local ethical committee (registration number DE/CA73/5550/09, Landesamt für Arbeitsschutz, Gesundheitsschutz und technische Sicherheit, Berlin, Germany). Informed written consent was obtained from each volunteer prior to the study. For the *in vivo* proof-of-concept study at 7.0 T, 3 healthy subjects without any known history of neuro- or cardiovascular diseases were included.

### Numerical EMF and Temperature Simulations in Phantoms and in a Human Voxel Model

For numerical simulations CST Microwave Studio (CST Studio Suite 2011, CST GmbH, Darmstadt, Germany) was used together with CST Design Studio for RF circuit co-simulations [31]. The thermal co-simulations were performed in CST MPhysics Studio solving the Bioheat transfer equation:
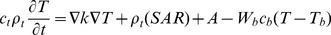
(1)with the specific heat of tissue 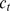, the tissue density 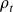, tissue temperature 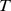, the thermal conductivity of tissue 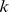, the basal metabolic heat rate 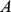, the blood perfusion rate 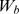, the specific heat of blood 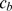 and the blood temperature 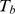. The mesh resolution was set below (2×2×2) mm^3^ for all simulations. To examine SAR and temperature distribution induced by constructive RF field interferences discrete ^1^H spin excitation frequencies at 1.5 T (64 MHz), 3.0 T (128 MHz), 7.0 T (298 MHz), 9.4 T (400 MHz), 11.7 T (500 MHz) and 14.0 T (600 MHz) were used.

**Figure 1:**
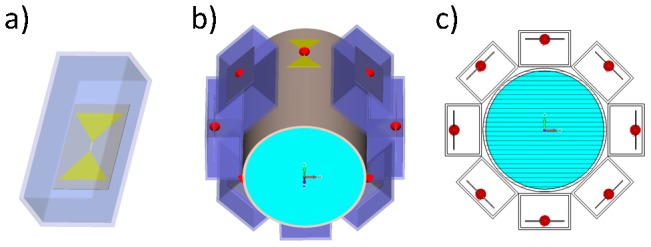
Basic design of the virtual antenna configuration used for electromagnetic field simulations. Basic design of the proposed bow tie dipole antenna building block used in numerical EMF simulations (**a**). Eight bow tie dipole antennas placed radially around a cylindrical phantom (**b**). Transversal view of the virtual phantom setup together with the bow tie dipole antennas (**c**).

Eight RF transmission channels – each with independent control of amplitude and phase – were employed. For each RF channel a bow tie dipole antenna design (Figure 1a) was used for transmission. Dipole antennas have been previously used for low temperature (∼42–45°C) hyperthermia applications [32]. RF characteristics and SAR performance of dipole antennas used for diagnostic MRI at 7.0 T were recently scrutinized [33]. The proposed bow tie antenna elements were positioned equidistantly and radially around a virtual cylindrical object (diameter = 172 mm, length = 250 mm) as indicated in Figure 1b–c. For the cylindrical object conductivity and permittivity that resembles brain tissue were used (σ_1_ = 0.657 S/m, ε_1_ = 50.5) [34], [35]. To shorten the effective length of the dipole antennas at the frequencies used the antennas were immersed in distilled water with a high relative permittivity constant of ε≈81 and a low conductivity of 0.065 S/m to reduce absorption losses. The width and length of the antennas at the frequencies used were derived from [36] and are surveyed in Table 1. Matching and tuning was performed with a match and tune network at the antennas feeding point calculated in an S-parameter analysis in RF circuit co-simulations.

**Table 1:** Synopsis of the excitation frequencies and antenna dimensions used for electromagnetic field simulations.

**Magnetic field strength [T]**	1.5	3.0	7.0	9.4	11.7	14.0
**Excitation frequency [MHz]**	64	128	298	400	500	600
**Bow tie length [mm] (triangle height)**	200	120	30	22.5	17.5	12.5

Dimensions of the bow tie antennas used for numerical EMF simulations. Magnetic field strengths ranging from 1.5 T (64 MHz) to 14.0 T (600 MHz) were applied. This approach was used to investigate specific absorption rate (SAR) distribution as a function of the excitation frequency.

To create a SAR focus due to constructive interferences of E-fields in the center of the phantom, all ports were excited in-phase (no phase shift between elements) with an accepted input power of P_in_ = 1 W (reflected power is not included) at the ports. The effective dimensions of the deep lying hotspots were analyzed using iso-contour calculations of the SAR distribution. For this purpose iso-SAR 25%, iso-SAR 50%, iso-SAR 75% and iso-SAR 90% thresholds were derived based on the maximum point SAR value.

Temperature simulations were performed at 298 MHz using the parameters found in the experimental setup with a background temperature of 20°C and an input power of 50 W per channel. To simulate the effect of RF heating over a three minute time period, the temperature was calculated based on the power loss distribution of an in-phase phase setting (Ch1-8: 0°). This setup yielded a deep lying hotspot in the center of the phantom. To demonstrate 2D hotspot steering RF heating over two minutes using a specific set of phases (Ch1: 0°, Ch2:45°, Ch3:180°, Ch4:225°, Ch5:0°, Ch6:225°, Ch7:135°, Ch8:45°) for the eight elements was applied.

To show the feasibility of targeted RF heating in the human brain, temperature simulations in the voxel model “Ella” derived from a healthy volunteer [37] were performed. For this purpose a dielectric medium with tissue equivalent properties (ε = 50, σ = 0.6 S/m) was used i) to improve coupling of the electromagnetic waves for each RF transmission channel to the head and ii) to cool down the surface of the head using a cooling temperature of 20°C. For this setup the input power was adjusted to 8×50 W with an in-phase phase setting (Ch1-8: 0°) that was customized to focus the E-fields in the center of the brain. The duration of the simulation was set to 5 minutes.

### Implementation of the Hybrid Applicator at 7.0 T

**Figure 2:**
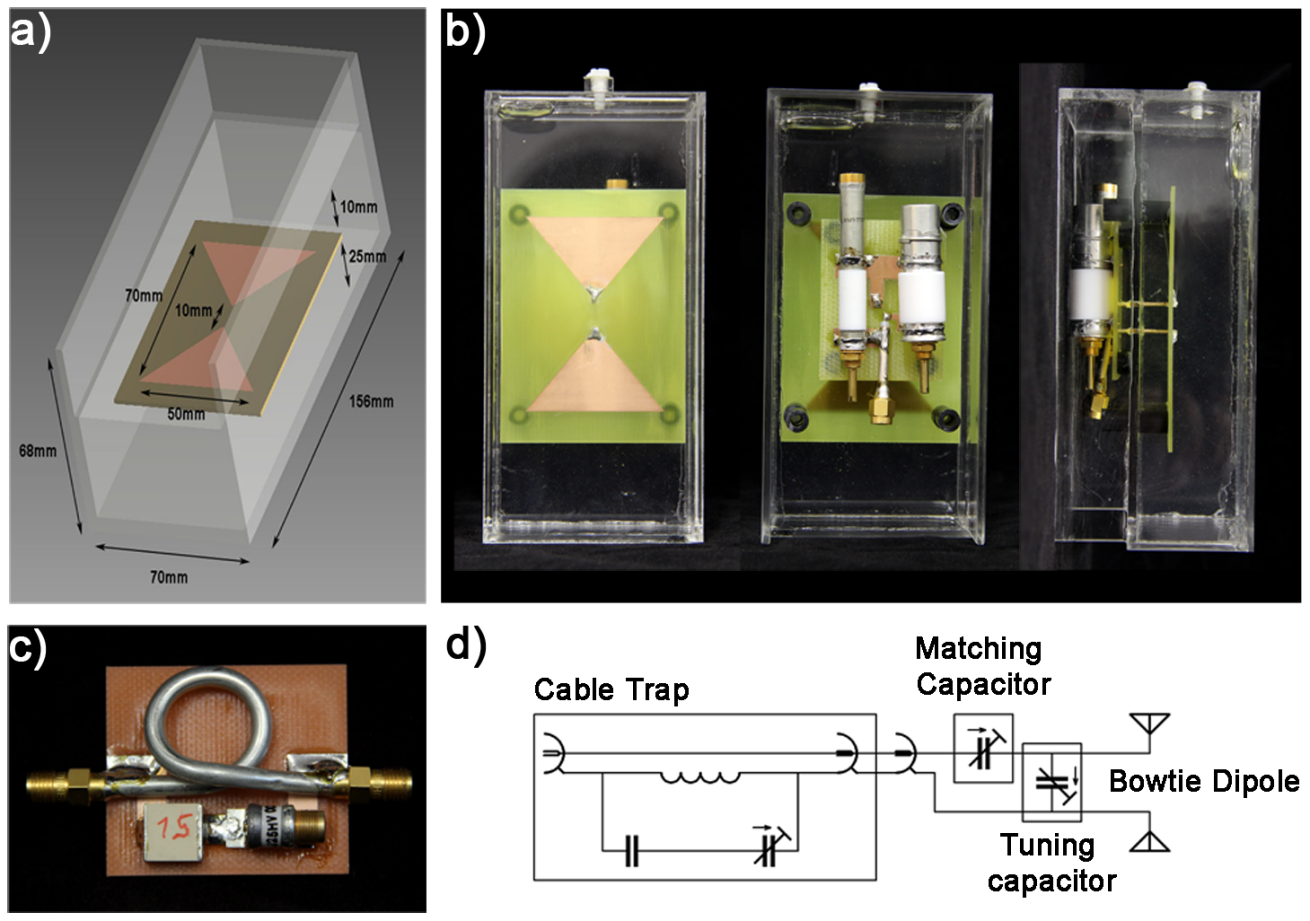
Experimental version of the bowtie antenna used in the hybrid applicator. Basic design and dimensions of the bow tie dipole building block used for MR imaging, MR thermometry and RF heating at 7.0 T (**a**). Picture photographs taken from the front, back and side of the bow tie antenna building block (**b**). Picture photograph of the cable trap design using semi rigid cable. Schematic diagram of the matching and tuning network connected to the antenna (**d**).

A bow tie antenna building block with the dimensions of (156×70×68) mm^3^ was built and adjusted to the 7.0 T MR frequency (298 MHz). Figure 2a–b show detailed views of the bow tie antenna building block. A bow tie design was chosen due to its increased 3 dB bandwidth of 143 MHz versus the 78 MHz bandwidth of a 10 mm rectangular strip dipole. This offers the advantage of an improved object specific tuning and matching, which favors inter-subject applications of the antennas together with an improved power transmission stability due to changing loading conditions like body movement. For the substrate the high permittivity medium Deuteriumoxide (D_2_O, isotopic purity 99.9 atom % D, Sigma Aldrich GmbH, Munich, Germany) was used. This allows smaller antenna dimensions due to a high refractive index of approximately 9. The gyromagnetic ratio of deuterium deviates from hydrogen and hence produces no signal at the ^1^H TX/RX frequency. This approach helps to acquire images free of artifacts caused by signal contributions from ^2^H deuteron substrate. A substrate that can act as a solvent benefits from an increased flexibility to change its permittivity. It also offers means for surface cooling, a feature beneficial for targeted RF heating interventions. The bow tie antenna was immersed in D_2_O substrate inside a polymethylmethacrylat (PMMA) cover with the dimensions of (40×150×70) mm^3^. From the antenna tip a parallel transmission line was connected to the matching and tuning network, which is located outside of the PMMA box (Figure 2b). To cope with a high power and voltage, highly voltage-rated nonmagnetic trimmers (Voltronics, Salisbury, USA) were used. The antennas and the matching and tuning network were realized on a printed circuit board (PCB) to allow reasonable reproducibility of the electromagnetic behavior between elements. For each element a cable trap – each consisting of a single loop cable, a fixed capacitor and a variable capacitor - was placed in the feeding cable creating a tuned parallel resonant circuit (Figure 2c). This approach imposes large impedance to signals conducted on the shield of the coax cable for a resonance frequency of 298 MHz. Coaxial semi rigid cables were used to guarantee 50 Ohm impedance conditions of the cable trap and to avoid excessive heating with the given power throughput. The basic scheme of the circuit used for a bow tie dipole element together with the matching and tuning network and the cable trap is depicted in Figure 2d.

**Figure 3:**
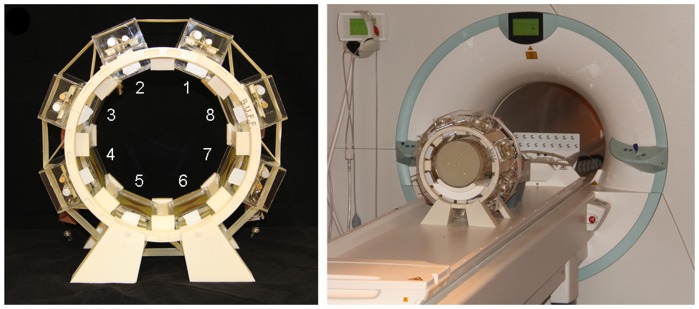
Experimental setup of the hybrid applicator used at a magnetic field strength of 7.0 T. Picture photograph of the eight channel TX/RX hybrid applicator implemented at 7.0T together with annotations that induce the transmission channel number (**left**). Picture photograph of the experimental setup which uses the hybrid applicator together with a cylindrical phantom at 7.0T (**right**).

For the hybrid multichannel applicator eight bow tie elements were placed in an equidistant radial pattern in a stereotactic holder. For accurate placement of the eight antennas the holder was created using a 3D computer aided design (CAD) model developed with Autodesk Inventor 2011 (Autodesk Inc., San Rafael, CA, USA). The holder was plotted with a 3D rapid prototyping system (BST 1200 es, Dimension Inc., Eden Prairie, MN, USA) using ABS+ material. Figure 3 illustrates the final setup of the 8 channel hybrid TX/RX applicator tailored for MR imaging, MR thermometry and targeted RF heating in a 7.0 T environment.

### Phantom Design

To validate EMF simulations versus MR measurements and to perform targeted RF heating experiments, a cylindrical phantom (length = 250 mm, diameter = 180 mm, wall thickness = 4 mm, polymethylmethacrylate (PMMA)) containing agarose gel (20 g/l) doped with NaCl (3.33 g/l) and CuSO_4_ (0.74 g/l) was built. NaCl was chosen to adjust the conductivity. CuSO_4_ doping was used to shorten T_1_ to approximately 300 ms to facilitate short repetition times for fast MR temperature measurements. Agarose was used to mimic heat conductivity and heat capacity of tissue. It was also chosen to prevent heat transfer due to convection. The medium exhibited a permittivity of ε = 75 and conductivity of σ = 0.72 S/m as measured with a network analyzer (Agilent 4296B, Santa Clara, California, USA) following a procedure published previously [38]. Four polyethylene terephthalate (PET) tubes were included in the gel to accommodate fiber optic thermo sensors used for temperature measurements independent of MRTh.

### Safety Assessment for MR Imaging

For targeted RF heating an input RF power that exceeds the clinical standards given by the IEC guidelines was applied. For *in vivo* MR imaging however, the energy deposition in tissue was limited to the values proposed by the IEC 60601-2-33 Ed.3. guidelines [30] to guarantee a safe application of the transmitted electromagnetic (EM) fields. Numerical SAR (10 g average) calculations were performed together with the voxel models “Duke” and “Ella” from the Virtual Family [37], as illustrated in Figure 1d. Whole body SAR, partial body SAR and local SAR values were evaluated and the power limits were set accordingly.

### Experimental Setup

All measurements were performed on a 7.0 T whole body MR system (Magnetom, Siemens Healthcare, Erlangen, Germany). For MR imaging, MRTh and targeted RF heating a set of eight power amplifiers (Stolberg HF Technik AG, Stolberg-Vicht, Germany) – each offering 1 kW maximum peak power together with independent control of phase and amplitudes - were connected with the eight channel dipole antenna elements of the hybrid applicator. For this purpose the applicator was connected to the MR system via a coil interface comprising 8 TX/RX switches and low-noise preamplifiers (Stark Contrasts, Erlangen, Germany).

Relative temperature measurements were performed using the proton resonance frequency shift (PRFS) method [39] with a dual gradient echo (GRE) technique [40], [41]. MR thermometry imaging parameters were: TE_1_ = 3 ms, TE_2_ = 10.14 ms, TR = 70 ms, slice thickness 6.0 mm, FOV = (300×300) mm^2^, in-plane spatial resolution (0.59×0.59) mm^2^, transmit reference voltage (per channel) U_ref_ = 100 V, nominal flip angle 40^0^, receiver bandwidth = 445 Hz/pixel, acquisition time 4.4 s. All temperature maps were acquired with an in-phase phase setting (0° phase shift between TX/RX elements). Changes of the static magnetic field over time (approximately 0.02 ppm/h) influence the measured proton chemical shift and lead to errors of the PRFS method of ±2 K (assuming a temperature coefficient of −0.01 ppm/K for the phantom). To account for these errors, the B_0_ phase drift was measured inside a vegetable oil sample, which was placed outside of the phantom throughout the experiments [42]. The phase drift inside the oil reference, which has a negligible temperature dependent chemical shift, was averaged over all pixel, excluding pixels close to the boundary of the sample to avoid incorrect phase contributions induced by susceptibility gradients at the oil/acryl interface.

For absolute temperature measurements and for validation of the MR thermometry maps, four optical thermo sensors were employed (OmniFlex, Neoptix, Quebec, Canada). Temperature calibration measurements were performed to scrutinize the accuracy of the MRTh method, yielding an experimental absolute error of ±1 K and a relative error of ±0.2 K for the fiber optic approach and ±2 K for MRTh.

To apply appropriate RF power essential for RF heating, a rectangular pulse with a pulse duration of 4 ms was used together with a TR of 32 ms and an amplitude of U = 170 V. This setup generates a duty cycle of 13% and an average power of approximately 72 W per transmission channel. Cable losses of around 30% lead to an average power of 50 W at each antenna. Antenna losses were not taken into account.

Two phase settings were used for the assessment of the applicator:

- All elements in-phase (0° phase shift between channels) to induce a SAR and temperature hotspot in the center of the phantom.
- A phase setting to demonstrate the feasibility of 2D steering of the SAR and temperature hotspot.

The phase settings used for RF heating were derived from numerical E-field simulations. For phase setting i) the heating period was 180 s followed by the acquisition of the temperature maps using the hybrid applicator. For phase setting ii) the heating period was 120 s followed by the acquisition of the temperature maps using the hybrid applicator.

For imaging, the transmit field efficiency 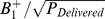 was evaluated and validated with EMF simulations. For this purpose B_1_^+^maps were acquired in the phantom using the Bloch Siegert method [43] in conjunction with a slice selective 2D gradient echo technique. The acquired B_1_^+^ maps were compared with the B_1_^+^ maps deduced from the numerical EMF simulations. For human brain imaging B_1_^+^ maps were acquired for each channel to gain a better insight into the transmit fields inside a heterogeneous object. This set of B_1_^+^ maps was used for slice selective B_1_^+^ shimming using the parallel TX PulseDesign Suite (Siemens Healthcare, Erlangen, Germany) with the goal of improving B_1_^+^ uniformity across an axial slice of the volunteer's brain.

To examine the parallel imaging performance of the hybrid applicator, geometry factor (g-factor) maps were determined using acceleration factors of R = 2, R = 3 and R = 4 together with GRAPPA reconstruction (32 calibration lines) [44]. For this purpose the noise of every element was measured *in vivo* using a noise prescan [45]. A noise correlation matrix was calculated.

## Results

### Numerical EMF Simulations from 1.5 T to 14.0 T

**Figure 4:**
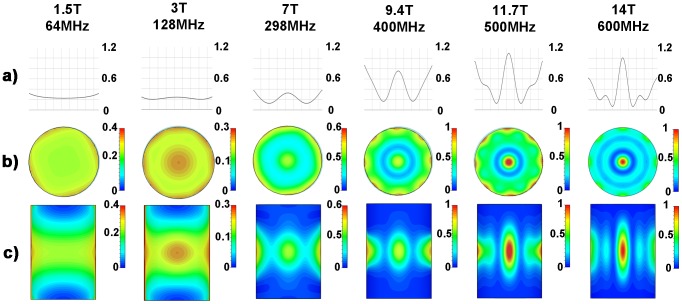
Synopsis of SAR simulations for frequencies ranging from 64 MHz (1.5 T) to 600 MHz (14.0 T). Point SAR [W/kg] distributions derived from numerical EMF simulations of an 8 channel bow tie antenna applicator using discrete MR frequencies ranging from 64 MHz (1.5 T) to 600 MHz (14.0 T). Point SAR profile along a middle line through the central axial slice of the cylindrical phantom (**a**). Point SAR distribution of the central axial slice of the cylindrical phantom (**b**). Point SAR distribution of the mid-coronal slice through the cylindrical phantom (**c**). A decrease in the size of the SAR hotspot was found for the axial and coronal view when moving to higher field strengths.

SAR distributions derived from numerical EMF simulations using discrete ^1^H MR frequencies ranging from 64 MHz to 600 MHz are illustrated in Figure 4. The SAR hotspot dimensions obtained for all frequencies are surveyed in Table 2 for a central axial slice through the phantom. At 64 MHz a rather uniform SAR distribution over the cylindrical phantom was observed. At 128 MHz focal regions of SAR increase were found which confirms results obtained for RF hyperthermia frequencies (f<140 MHz) used in a clinical setting. For this frequency the iso-SAR 90% region located in the central axial slice through the phantom exhibits a circular shape with a diameter of 59 mm. However, at this frequency the iso-SAR 25%, the iso-SAR 50% and the iso-SAR 75% contour lines encompass the entire central axial slice with additional iso-SAR 90% side lobes at a depth of 8 mm distance from the phantoms surface. When moving to higher frequencies/shorter RF wavelengths the size of the focal hotspot area decreased as demonstrated in Figure 4. Also, the power deposition inside the phantom per input power (SAR_center_/P_in_) increased (Figure 4a) making targeted RF heating more efficient. At 7.0 T (298 MHz) the E-field focusing abilities of the dipole antenna array yielded an iso-SAR 50% hotspot with a diameter of 43 mm. The SAR hotspot was even further reduced at 14.0 T (600 MHz). Here the iso-SAR 90% contour covered a circular area with a diameter as small as 10 mm for an axial slice drawn through the center of the phantom. In comparison the iso-SAR 75% contour included a diameter of 17 mm, while the iso-SAR 50% and iso-SAR 25% diameter revealed a value of 26 mm and 35 mm for a central axial slice through the phantom. At a frequency of 600 MHz no iso-SAR 90% and iso-SAR 75% were found to be present at the surface of the phantom. The iso-SAR 50% encapsulates a distance of 5 mm from the surface and the iso-SAR 25% runs at a distance of 18 mm from the phantoms surface. This behavior leads to rather low surface SAR values compared to the center of the phantom.

**Table 2:** Synopsis of the specific absorption rate distribution derived from electromagnetic field simulations.

	Center SAR				Surface SAR			
Excitation frequency [MHz]	iso-SAR 90% [mm]	iso-SAR 75% [mm]	iso-SAR 50% [mm]	iso-SAR 25% [mm]	iso-SAR 90% [mm]	iso-SAR 75% [mm]	iso-SAR 50% [mm]	iso-SAR 25% [mm]
64	-	-	O	O	10	40	O	O
128	59	O	O	O	8	O	O	O
298	-	-	43	O	1	5	17	O
400	-	20	38	59	5	13	27	41
500	12	20	32	44	1	6	16	44
600	10	17	26	35	-	-	5	18

Specific absorption rate (SAR) hotspot diameter in the axial plane for iso-SAR 90%, iso-SAR 75%, iso-SAR 50% and iso-SAR 25% contour lines obtained from EMF simulations using discrete MR frequencies ranging from 1.5 T (64 MHz) to 14.0 T (600 MHz). (O) indicates that the whole object is included in the given iso-SAR contour. (−) indicates that no such iso-SAR value was found in the given ROI.

### Implementation of the Hybrid Applicator at 7.0 T: Imaging Characteristics

**Figure 5:**
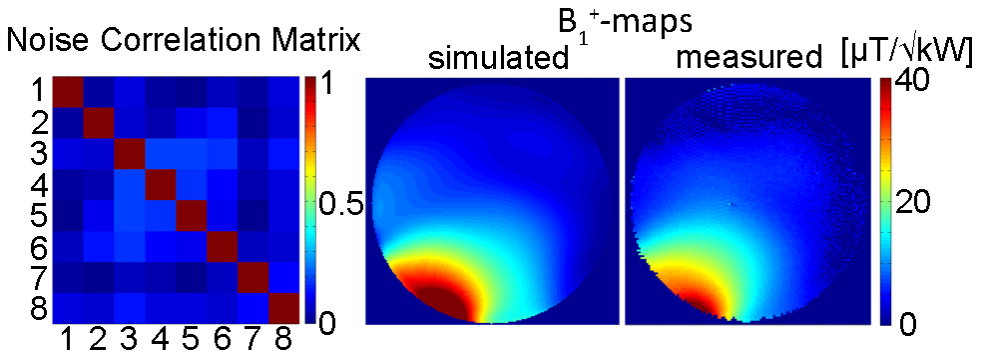
RF performance of the experimental hybrid applicator. Noise correlation matrix obtained for the decoupling of the 8 elements included in the proposed 8 channel TX/RX applicator (**left**). Simulated B_1_^+^-map in [µT/√kW] derived from a single element; channel 5 in this case (**middle**). For this purpose a transversal slice through the center of the phantom was used. For comparison the measured B_1_^+^-map is shown [µT/√kW] for the same slice and bow tie antenna element (**right**).

Matching and tuning parameters were below −25 dB. Decoupling between elements was found to be below −21 dB in the phantom setup. Noise correlation (*in vivo*) was 0.16±0.09 (mean ± std) for all elements with a maximum measured value of 0.36 between element 6 and element 8. Figure 5 shows a noise correlation matrix that indicates a rather low noise correlation and a reasonable decoupling between elements which is essential for parallel imaging. For phantom studies a match between the simulated and the measured B_1_^+^ maps was obtained as illustrated in Figure 5. B_1_^+^ mapping yielded a B_1_^+^ of 8.2 µT/√kW in the center of the phantom and a B_1_^+^ of 42 µT/√kW at the phantoms surface. In comparison, EMF simulations revealed a B_1_^+^ of 8.2 µT/√kW in the center and a B_1_^+^ of 59 µT/√kW at the surface of the phantom.

**Figure 6:**
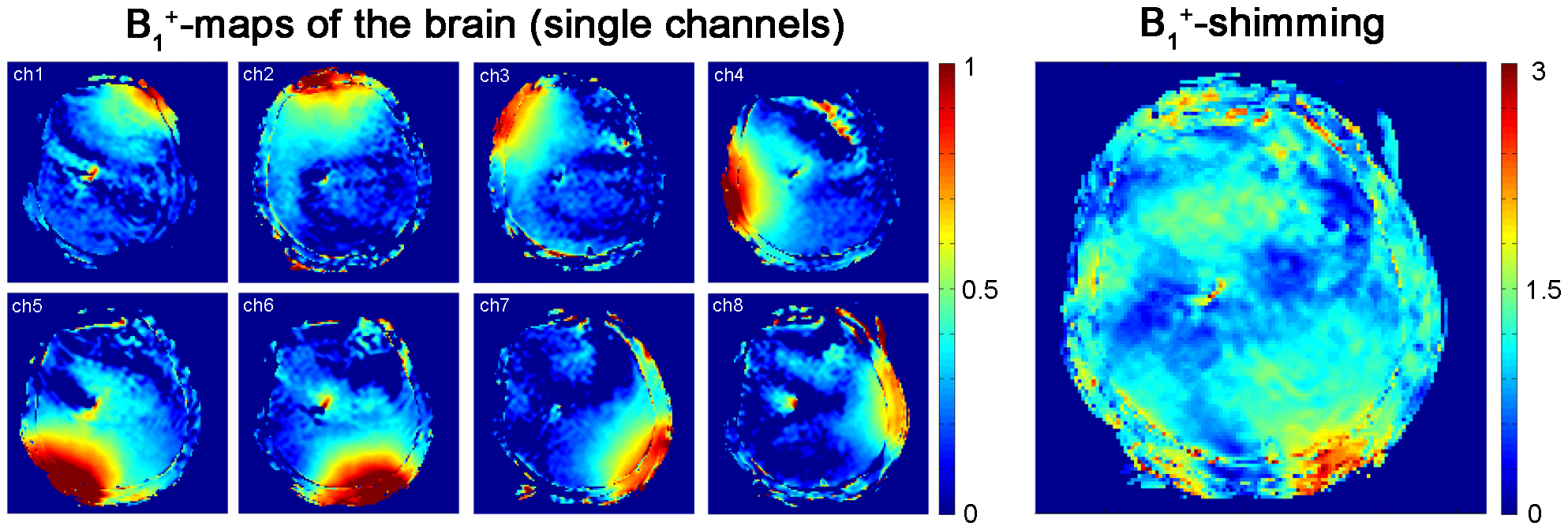
Transmission fields (B_1_^+^) of the hybrid applicator at 7.0 T in the human brain. *In vivo* brain B_1_^+^ maps obtained from Bloch Siegert mapping of the eight independent channels of the applicator (**left**). For B_1_^+^ mapping an axial slice through the subject's brain was used. The colour scale is in units of 16 µT/√kW. B_1_^+^map of the volunteers brain after B_1_^+^ shimming (**right**). The B_1_^+^map shows rather uniform B_1_^+^distribution.

**Figure 7:**
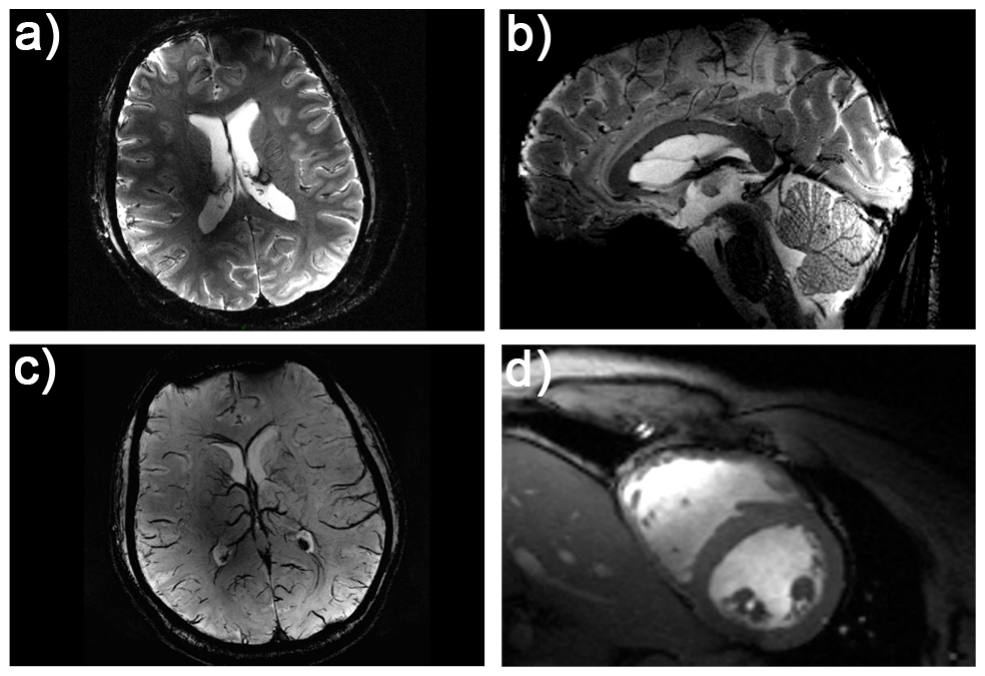
*In vivo* imaging of the human brain and the human heart using the bow tie antennas. Illustration of the imaging capabilities of the hybrid TX/RX applicator driven by bow tie antennas. High spatial resolution MR images of the human brain (**a, b**). A gradient echo technique was used with a spatial resolution of: (0.5×0.5×2.0) mm^3^, FOV = (200×175) mm^2^, TR = 989 ms, TE = 25 ms, reference transmitter voltage U_ref_ = 170 V, nominal flip angle = 35°, receiver bandwidth = 30 Hz/pixel. Minimum intensity projection derived from susceptibility weighted 3D gradient echo imaging of the human brain (**c**). Imaging parameters: spatial resolution: (0.5×0.4×1.2) mm^3^, FOV = (184×184) mm^2^, TR = 25 ms, TE = 14 ms, reference transmitter voltage U_ref_ = 170 V, nominal flip angle = 24°, 16 slices per slab, receiver bandwidth = 120 Hz/pixel, flow compensation. Short axis view of the human heart (**d**). Images were acquired using a 2D CINE FLASH technique, FOV = (360×326) mm^2^, TE = 2.7 ms, TR = 5.6 ms, receiver bandwidth = 444 Hz/px, 30 cardiac phases, 8 views per segment, slice thickness 4 mm, spatial resolution: (1.4×1.4×4) mm^3^, nominal flip angle = 35°, reference transmitter voltage U_ref_ = 400 V.

*In vivo* B_1_^+^ maps derived from B_1_^+^mapping of each element are depicted in Figure 6 for a mid-axial slice of the brain. For comparison B_1_^+^ maps deduced from EMF simulations using the calculated B_1_-shim setting are shown in Figure 6. B_1_^+^ shim optimization revealed transmitter phases of 69° (Ch1), 156° (Ch2), 74° (Ch3), 129° (Ch4), 92° (Ch5), 0° (Ch6), 276° (Ch7) and 147° (Ch8). This phase setting yielded an average B_1_^+^ of 17.2 µT/√kW over the whole mid-axial slice of the human brain with a standard deviation of 6.2 µT/√kW. This subject specific B_1_^+^ shim was used for gradient echo imaging of the brain at 7.0T as shown in Figure 7. The assessment of the hybrid applicators parallel imaging performance revealed averaged g-factors of 1.2±0.1 for R = 2, 1.7±0.4 for R = 3 and 2.7±0.7 R = 4 for an axial slice through the brain.

### RF Heating Using the Hybrid TX/RX Applicator at 7.0 T

**Figure 8:**
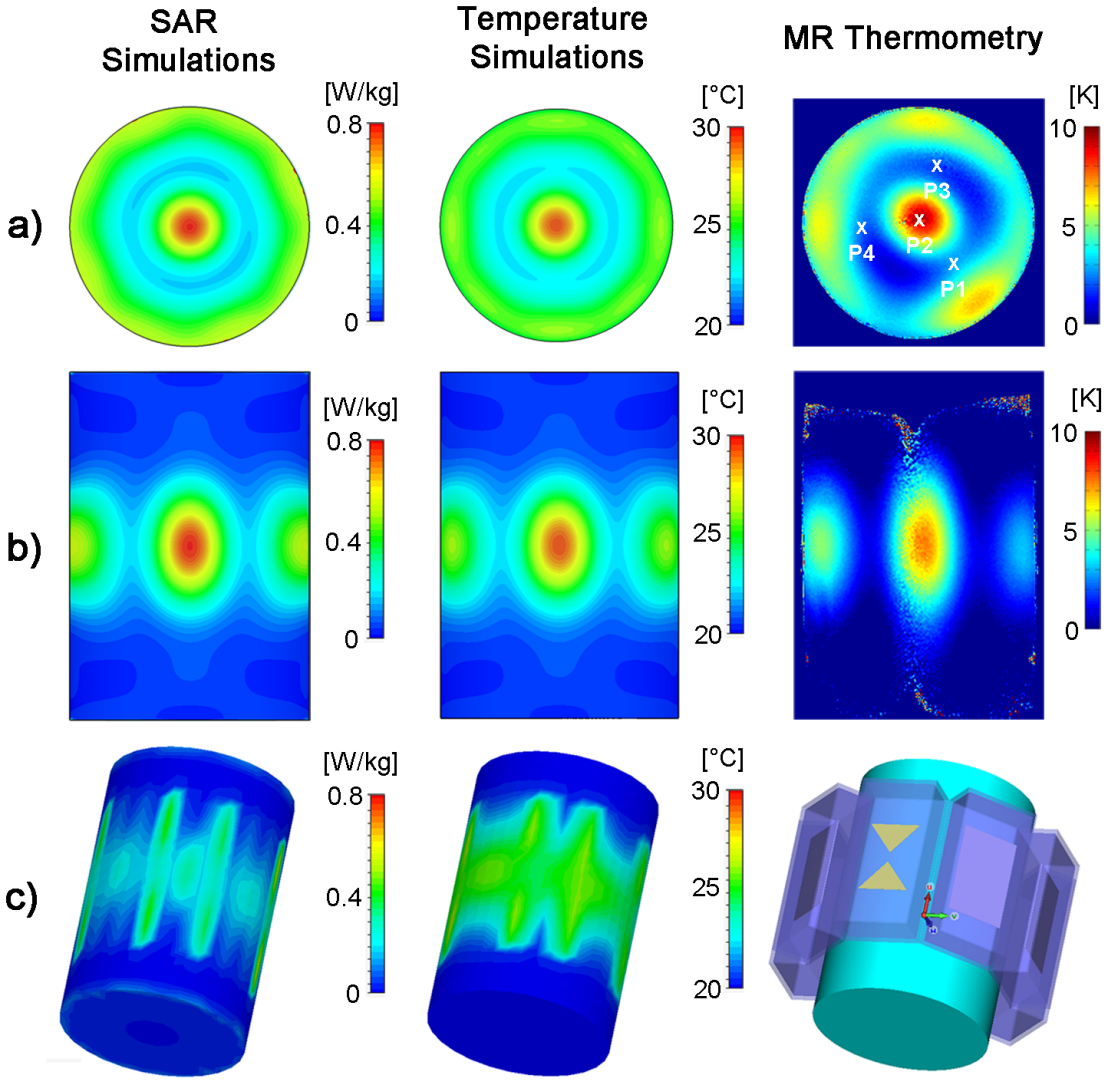
Targeted RF heating in a phantom: simulation and experiment. Axial and coronal views of specific absorption rate (**left**) and temperature (**middle**) distribution derived from EMF and temperature simulations using an 8 channel applicator together with a cylindrical phantom and a ^1^H excitation frequency of 298 MHz. For comparison, a temperature map derived from MR thermometry of the same slice at 7T (298 MHz) using the TX/RX applicator is shown (**right**). For the experimental setup a heating period of 3 min was used. SAR and temperature hotspots were induced in the center of the phantom by using no phase shift between the bow tie antennas. P1–P4 indicate the location of the fiber optic temperature probes.

**Figure 9:**
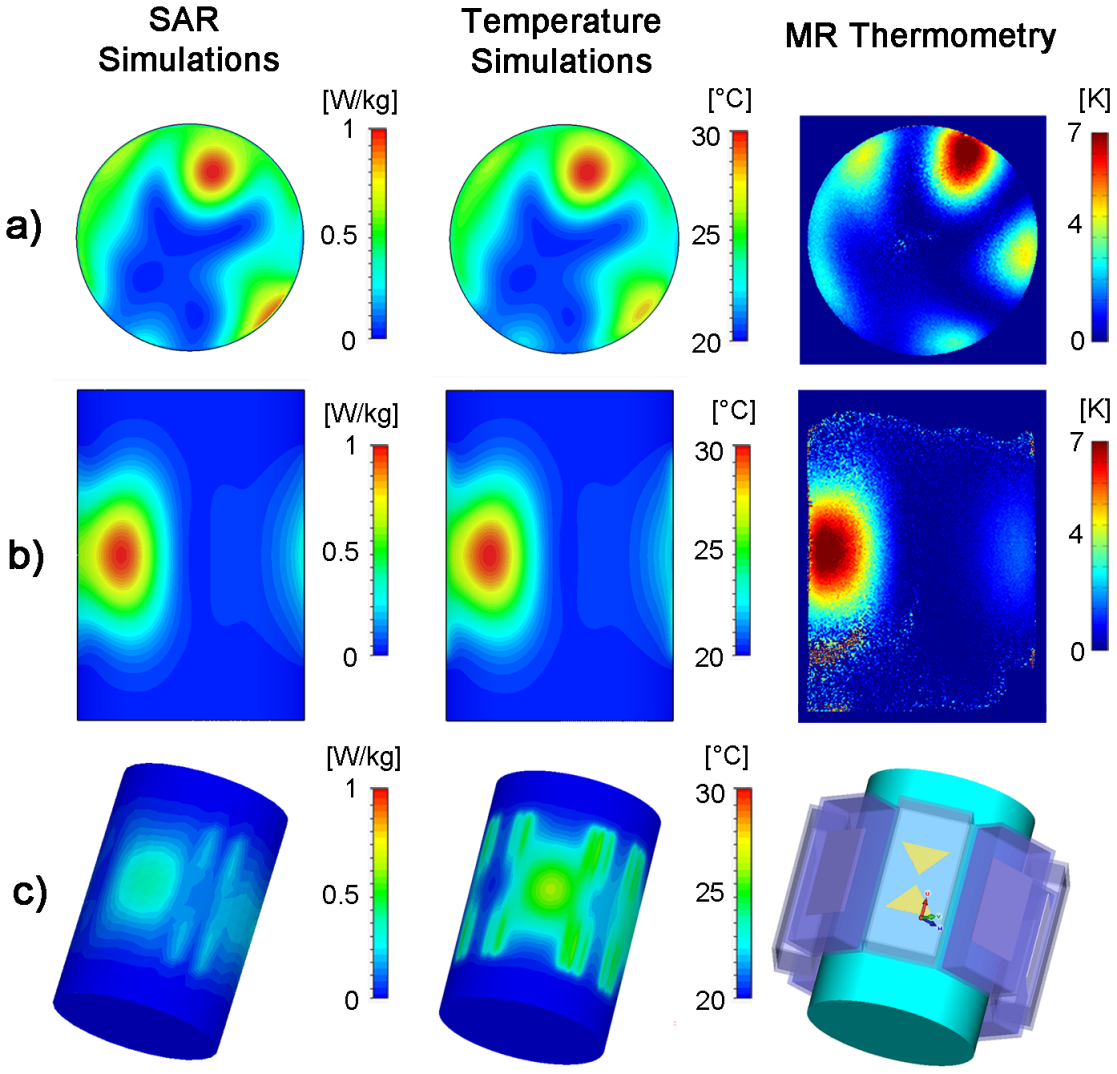
2D steering of targeted RF heating in a phantom: simulation and experiment. Axial and coronal views of specific absorption rate (**left**) and temperature (**middle**) distribution derived from EMF and temperature simulations using the 8 channel applicator, a cylindrical phantom and a ^1^H excitation frequency of 298 MHz. For comparison, a temperature map derived from MRTh acquisitions at 7T (298 MHz) using the TX/RX applicator is shown (**right**). For the experimental setup a heating period of 120 s was used. A set of phase shifts (Ch1:0°, Ch2:45°, Ch3:180°, Ch4:225°, Ch5:0°, Ch6:225°, Ch7:135°, Ch8:45°) between the bow tie antennas was used to steer the SAR and temperature hotspot towards the surface of the phantom.

Using the hybrid TX/RX applicator deep-seated SAR and temperature hotspots were generated in the phantom as demonstrated in Figure 8. The hybrid TX/RX applicator facilitates steering of the SAR and temperature hotspots via changes to the inputs of the elements to another location as depicted in Figure 9.

For a phase setting i) with all elements driven in-phase, the EMF simulations showed higher SAR values in the center of the phantom compared to the surface regions (Figure 8). The surface SAR in the agarose phantom didn't exceed a value of 0.52 W/kg. In comparison, the center of the phantom showed a value of 0.79 W/kg. The simulated SAR hotspot in the phantom yielded dimensions of (19×19×28) mm^3^ for iso-SAR 90%, (31×31×47) mm^3^ for iso-SAR 75%, (48×48×71) mm^3^ for iso-SAR 50% and (70×70×99) mm^3^ for iso-SAR 25%. For the temperature co-simulations the resulting temperature increase due to the calculated power loss distribution was ΔT = 11.6 K in the center and ΔT = 7.4 K at the surface of the phantom.

The RF heating experiments confirmed the predictions of the EMF simulations. MR temperature maps are shown in Figure 8. After a heating period of 180 s with approximately 50 W average power per channel, a maximum temperature increase of ΔT = 10.7 K (averaged value over 9 pixel) was obtained for the center of the phantom. The maximum temperature increase found for a surface region of the phantom was ΔT = 6.7 K (averaged over 9 pixel). The thermo fiber optical probes confirmed the findings derived from MRTh. After the heating period a temperature increase of ΔT = 9.6 K was observed at position P2 (Figure 8) in the center of the phantom. The three fiber optic sensors positioned 4.3 cm off-center yielded a temperature increase of ΔT = 3 K at position P1 versus a temperature increase of ΔT = 1.7 K at position P3 and ΔT = 2 K at position P4.

By changing the phase setting for each dipole antenna element the SAR and temperature hotspot was repositioned from the center of the phantom to a region close to the surface of the phantom. For this purpose the phase settings (Ch1:0°, Ch2:45°, Ch3:180°, Ch4:225°, Ch5:0°, Ch6:225°, Ch7:135°, Ch8:45°) derived from the EMF simulations were applied. This phase setting configuration induced a temperature increase in a region close to the phantoms surface as demonstrated in Figure 9. The simulations revealed a SAR value of 1.01 W/kg in the center of the SAR hotspot versus SAR = 0.96 W/kg at the surface of the phantom. This SAR behavior translated into a temperature increase of ΔT = 11.5 K in the center of the hotspot. The MRTh measurements revealed a max temperature increase of ΔT = 8.1 K in the hotspot after a heating period of 120 s as shown in the temperature maps in Figure 9.

**Figure 10:**
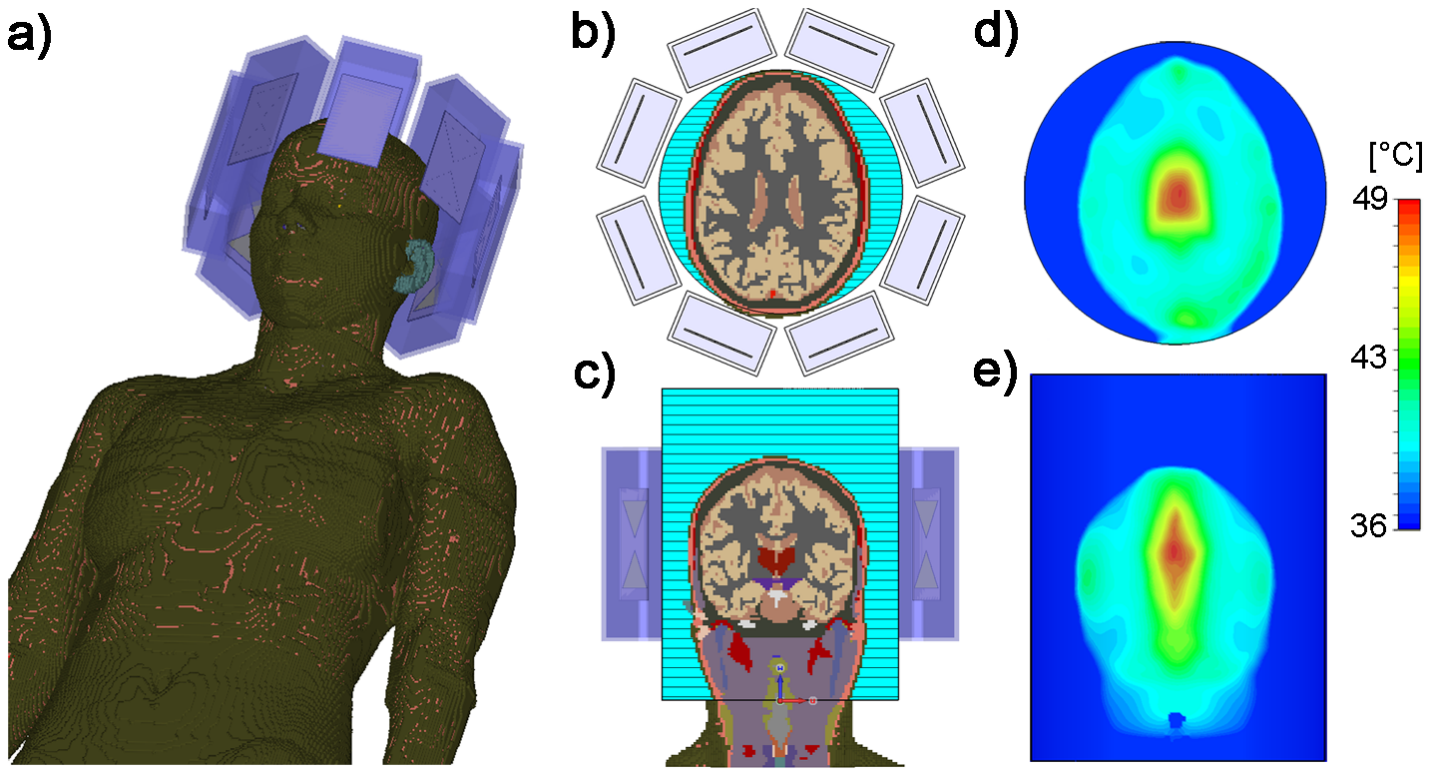
Simulation of RF heating in a human voxel model. Temperature simulations performed using the *in vivo* human voxel model “Ella” [37] in conjunction with the hybrid applicator. Positioning of the voxel model and eight bow tie dipole antennas (**a**). Axial and coronal slices through the human brain together with the dielectric medium adjusted to T = 20°C (**b–c**). Simulated temperature maps for a axial and coronal slice of the human brain (**d–e**). For this purpose RF heating was conducted over 5 min using an average RF power of 50 W per channel at 298 MHz. For the center of the brain the maximum temperature was 48.6°C upon completion of the RF heating paradigm (**d**). In comparison the cranium's surface did not exceed a temperature of 43.3°C for the same heating paradigm.

Temperature simulations in the human brain are depicted in Figure 10a–e). After a heating period of 5 min with an input power of 8×50 W, the temperature in the central hotspot was found to be 48.6°C. For comparison the cranium's surface did not exceed a temperature of 43.3°C. The deep-seated hotspot showed a size of (19×23×32) mm^3^ for iso-temperature 90%, (29×35×68) mm^3^ for iso-temperature 75% and (41×56×112) mm^3^ for iso-temperature 50%.

## Discussion

This study outlines the technical underpinnings of a hybrid transmit/receive applicator and demonstrates the basic feasibility of RF heating using the proposed applicator design together with EMF simulations conducted for discrete frequencies ranging from 1.5 T to 14.0 T. Our EMF simulations and experiments demonstrate the feasibility of an 8 channel TX/RX hybrid applicator for MR imaging, MR thermometry and controlled targeted RF heating at 7.0 T. The evaluated applicator utilizes the proton MR frequency for targeted RF heating and can be used together with commercially available MR systems and multi-channel transmit systems for diagnostic and interventional applications. Unlike previous approaches, where an MR system is combined with an extra RF heating setup running at a different frequency [9], [12], [13], the concept proposed here makes additional RF hardware (RF power amplifiers, RF electronics, filters, RF heating antennas) or software to drive these components dispensable. This truly hybrid approach makes furthermore use of its inherent local multi-channel RX elements, which increases SNR and enhances parallel imaging performance [46]–[48] with the goal of high spatial and temporal MR temperature mapping during RF heating interventions. It's high field use including field strengths of up to 14 T demonstrates higher heating efficiencies and reduced hotspot sizes for RF hyperthermia applications as compared to other low field (3T) approaches [49].

Our experimental results suggest that the proposed setup is capable of providing enough energy at 7.0T to heat up an elliptical area as small as (25×22×41) mm^3^ (simulated value: (31×31×47) mm^3^) for an iso-temperature 75% inside a uniform phantom with a maximum temperature increase of ΔT = 10.7 K within a 180 s heating period using an average power of 50 W per channel. In comparison, the temperature increase at the surface of the phantom was only ΔT = 6.7 K without using surface cooling. After showing proof-of-principle for focal radiofrequency heating of a hotspot in the center of the phantom we demonstrated the feasibility of steering a SAR/temperature hotspot to a surface location in the phantom. For this purpose a tailored set of excitation phases derived from EMF simulations was implemented for the applicators transmission elements. By using a human voxel model of a healthy volunteer our temperature simulations demonstrate that an RF induced hotspot inside the human brain can be generated using the proposed hybrid applicator at 7.0 T. After running an RF heating paradigm proposed here for five minutes a temperature increase to 48.6°C was accomplished in the center of the human brain. This approach underlines the importance of numerical simulations for SAR and temperature assessment in phantoms and *in vivo* RF heating interventions [50], [51]. Considering the MR magnet bore in the EMF simulation may further reduce the minor mismatch between the simulated and measured B_1_^+^ transmission fields and the temperature distributions. On the downside it should be noted that a resonant coupling of the antennas to the magnet bore increases radiation losses, decreases the antenna transmit efficiency and influences the field distribution inside the phantom [52]. A minor difference in the electric and thermic properties of the phantom and the antennas used in the simulations versus the experiments might present another potential source of error. A change of the z-dimension of the hotspot between phantom and in-vivo temperature simulations may arise from the geometrical differences of the cylindrical phantom and a sphere-like geometry of the human head, which influences the E-field vector orientation at its curved electromagnetic boundary.

On the MR physics and electrodynamics side the EMF simulations shown here provide an example on how the traits inherent to ultrahigh MR can be put to use beyond the common improvement in spatial resolution. The basic feasibility of targeted RF heating at MR frequencies of up to 600 MHz can be considered as an essential precursor for designing and building a hybrid applicator suitable for imaging and targeted RF heating at field strengths larger than 7.0 T. Admittedly, the clinical potential of RF heating interventions at 7.0 T and even higher magnetic fields is as yet untapped. To push the envelope of basic MR research we envision to progress towards an experimental implementation at 500 MHz (11.7 T) for transmission. Our results clearly indicate that higher frequencies show a potential benefit for targeted RF heating applications [53]. It could be shown that this is valid for discrete MR frequencies ranging from 1.5 T to 14.0 T. In particular, the ratio between the hotspot SAR and the surface SAR is enhanced for excitation frequencies f≥500 MHz which facilitates improvements in the RF heating capabilities.

The observation that the hotspot dimensions in the phantom are more focused when using higher frequencies has major implications for future hybrid applicator designs. The size of the antenna elements can be reduced significantly at higher frequencies. This reduction in antenna size would afford a placement of even more transmission elements around the area of interest. This approach would support the intention of spreading the surface SAR more evenly across the surface and would help to further increase the SAR_center_/SAR_surface_ ratio. An increase in the number of independent transmission elements - each with exquisite phase and amplitude control - would also be instrumental to further sharpen the geometry and size of the temperature hotspot.

The proof-of-concept study presented here is ultimately aiming at advancing the capabilities of UHF-MR guided RF heating procedures and interventional therapies. Interventions may include temperature driven targeted drug and contrast agent delivery in conjunction with diagnostic MR imaging and spectroscopy and MR temperature mapping control. It is also to be expected that the proposed ultrahigh field RF heating approach will help to further improve the treatment efficiency of today's RF hyperthermia interventions used in cancer therapy. For example, with the size of the hotspot being significantly decreased at ultrahigh fields versus today's 64 MHz and 100 MHz clinical implementations we envision RF hyperthermia being put to use not only for the treatment of abdominal and pelvic tumors but also offering the potential to be employed for RF heating interventions of brain tumors. In this context potential applications could also include targeted drug or stem cell delivery to the myocardium or other regions afforded by local RF heating. One could even conduct a thought experiment where targeted RF heating driven by multitransmit UHF-MR technology is used for RF ablation versus today's invasive intracardiac catheter ablations as proposed in a recent review on the progress and promises of cardiac MR at ultrahigh fields [54].

The heavy water used to immerse the individual antennas showed excellent properties for a hybrid applicator. This approach affords low RF losses, negligible background signal from the antennas and small antenna size due to a high permittivity. Also, the fluid properties of the substrate enable a broad range of improvements for the traditional setup. For example it supports the use of a water bag that fits to the geometry of the target body section. This approach is thought to further improve efficiency of RF transmission to the patient and to enhance the imaging and heating properties. A cooling mechanism of the surface using heavy water circulation can be employed to dissipate undesired heat from surface regions.

It is a recognized limitation of this feasibility study that only 2D steering has been used to move the SAR and temperature hotspot to an arbitrary position in the phantom. For this reason we anticipate an arrangement of bow tie antennas not only in the axial plane, but also along the direction of the main magnetic field (z-axis) to enable 3D steering capabilities of the SAR/temperature hotspots. These efforts will be paralleled by moving towards a heterogeneous head phantom, which would enable a more realistic model for the assessment of thermal distributions. For this purpose we anticipate to position/design the antennas in such a way, that the Poynting vector is perpendicular to the electromagnetic boundary layer (cranium in case of the human brain) and directed towards the targeted region of interest. Such an arrangement with a directed EM energy towards the focus point, while more realistic, will potentially reduce the 3D hotspot dimension in z-direction as compared to the cylindrical phantom setup used in this study.

Our results may inspire further research to gain a better insight into the effect of RF pulse sequences on temperature elevation for a given time-average SAR [55] together with system and SAR characterization of parallel RF transmission [56]. Our work also suggests further innovations for directly measuring and monitoring E-fields [57]–[59], temperature changes induced by the radiofrequency fields in interventional MRI [60] as well as developments of B_1_^+^ phase mapping techniques at ultrahigh fields and its application for *in vivo* electrical conductivity and permittivity mapping [61]. Driving the proof-of-principle demonstrated in this study closer to the clinical scenario requires real time feedback capabilities to manage temperature measurements and RF power/RF control simultaneously [23].

To summarize, the opportunities and capabilities of ultrahigh field MR for RF heating based interventions shown here are intriguing and in a creative state of flux. Bringing ultrahigh field RF heating interventions and therapies into the clinic remains a major challenge and remains to be researched further.
